# Is the quality of evidence in health technology assessment deteriorating over time? A case study on cancer drugs in Australia

**DOI:** 10.1017/S0266462323000259

**Published:** 2023-05-18

**Authors:** Yuan Gao, Mah Laka, Tracy Merlin

**Affiliations:** Adelaide Health Technology Assessment, School of Public Health, University of Adelaide, Adelaide, SA, Australia

**Keywords:** health technology assessment, cancer medicines, risk of bias, evidence quality, reimbursement, evidence-based policy, decision-making

## Abstract

**Objective:**

This study aimed to assess whether there have been changes in the quality of clinical evidence submitted for government subsidy decisions on cancer medicines over the past 15 years.

**Methods:**

We reviewed public summary documents (PSDs) reporting on subsidy decisions made by the Pharmaceutical Benefits Advisory Committee (PBAC) from July 2005 to July 2020. Information was extracted on the study design, directness of comparison, sample size, and risk of bias (RoB). Changes in the quality of evidence were assessed using regression analysis.

**Results:**

Overall, 214 PSDs were included in the analysis. Thirty-seven percent lacked direct comparative evidence. Thirteen percent presented observational or single-arm studies as the basis for decisions. Among PSDs presenting indirect comparisons, 78 percent reported transitivity issues. Nearly half (41 percent) of PSDs reporting on medicines supported by head-to-head studies noted there was a moderate/high/unclear RoB. PSDs reporting concerns with RoB increased by a third over the past 7 years, even after adjusting for disease rarity and trial data maturity (OR 1.30, 95% CI: 0.99, 1.70). No time trends were observed regarding the directness of clinical evidence, study design, transitivity issues, or sample size during any of the analyzed periods.

**Conclusion:**

Our findings indicate that the clinical evidence supplied to inform funding decisions for cancer medicines is often of poor quality and has been deteriorating over time. This is concerning as it introduces greater uncertainty in decision making. This is particularly important as the evidence supplied to the PBAC is often the same as that supplied to other global decision-making bodies.

## Introduction

In Australia, three out of every ten deaths are attributed to cancer, with over 1 million people diagnosed with cancer every year (1). This high disease burden results in high costs. From 2017 to 2018, 32 percent of the Australian Government’s expenditure on medicines was spent on antineoplastic and immunomodulating agents, which was the highest among all government-funded medicines (2). To deliver effective, safe, and affordable health care to the public, new anticancer drugs are evaluated by the Pharmaceutical Benefits Advisory Committee (PBAC) before being listed on the Pharmaceutical Benefits Scheme (PBS) (3). PBAC is the health technology assessment (HTA) decision-making body that provides scientific advice to the Australian Government on the clinical effectiveness and cost-effectiveness of new medicines (3). Dossiers of clinical and economic evidence are supplied to the PBAC by sponsors of new medicines and these are independently evaluated by academic HTA groups to inform PBAC decision making. The outcomes of PBAC deliberations are published in the form of public summary documents (PSDs) (3).

Recently, Mintzes et al. found that regulatory approvals of new cancer drugs were underpinned by flawed evidence (4). Medicines are reaching the market without being tested in randomized controlled trials (RCTs) (5;6), and about half of this direct evidence has a high risk of bias (RoB) (6). In addition, there is an increase in the utilization of surrogate outcomes in oncology research (6;7), while the association between surrogates and true clinical benefits has been questioned (8;9). A lack of direct comparative evidence and the utilization of surrogate outcome measures results in increased uncertainty about the clinical benefits and risks associated with medicines (10). Although the evidentiary requirements are often different between regulators and payers, with the former focusing on the medicines’ risk–benefit to patients and the latter concerned with the value for money, there is a concern that poorer quality evidence at market approval may also bleed into reimbursement decision making. Reimbursing cancer drugs with uncertain evidence of improving or extending quality of life may not deliver meaningful value to patients or society (11).

Two previous studies have expressed concerns about the quality of clinical evidence (12;13) when evaluating submissions proposed for listing on PBS. Hill and colleagues found a significant proportion of submissions had problems with estimation of comparative clinical efficacy, mainly resulting from poor quality trials, incorrect interpretation of trial results, and use of surrogate outcomes (12). Wonder and colleagues (13) reviewed PSDs from 2005 to 2012 and found that a large proportion of clinical evidence failed to support the reimbursement claims, especially in terms of medicines’ comparative performance.

There have been significant changes to HTA and PBAC evaluation in Australia in the last decade (14). To define the population that can benefit the most, an increasing number of companion tests have emerged that involve the targeting of cancer treatments on the basis of genetic biomarkers (15). In Australia, an HTA process was introduced to evaluate these tests and treatments simultaneously (16). In this case, the submissions that contained these technologies were called “codependent submissions” (16). Clinical evaluations of codependent technologies are more complex than the traditional submissions that only included clinical trials for cancer drugs (15), because the evidence of diagnostic tests is also taken into consideration. The inclusion of this evidence may have impacted the overall evaluation of the quality of evidence. Another significant change to HTA processes over this time period was the implementation of parallel processing of regulatory and reimbursement medicine submissions, which aimed to improve the speed of reimbursement. From 2011, this novel policy allowed drugs to be evaluated by Therapeutic Goods Administration (TGA) and PBAC concurrently (17). In this process, the PBAC decision about the funding of new cancer drugs would be released as soon as market authorization was granted by the TGA (17). It is possible that drugs under parallel review may have insufficient evidence because PBAC has to make a decision without the evidence generated during post-licensing periods.

In our study, we evaluated the quality of evidence on cancer medicines but assessed this from a broad perspective (2005–2020). We focused not only on the change in the quality of evidence but also on potential determinants of the quality of evidence, including codependent technologies and the TGA–PBAC parallel process. To date, no previous studies have assessed these aspects in Australia.

In this study, we had three main research questions:

1. What was the quality of evidence in cancer drug submissions to the PBAC?
2. What factors influence or predict the quality of evidence in cancer drug submissions to the PBAC?
3. Has the quality of evidence in cancer drug submissions to the PBAC deteriorated over time?

## Method

### Brief requirements of submissions to PBAC

When an application relating to a new medicine or vaccine, or a new patient indication for these, is submitted to the PBAC for consideration, the application has been historically called a major submission. These major submissions include information related to the context of proposed use of the medicine (population, intended use in PBS, main comparator), clinical evaluation, economic evaluation, and predicted uptake of the medicine in practice.

In the clinical evaluation, applicants are required to provide the best available evidence to support the claimed effectiveness and safety of the proposed medicine for the specific patient indication/s with reference to a comparator (14). The ideal main comparator should be the current alternative therapy used in Australian clinical practice to treat these patients, and the one that is most likely to be replaced. PBAC strongly prefers clinical and economic evaluations that are based on direct randomized trials (RCT), where the intervention (the cancer drug) is compared to the main comparator in the same trial population.

### Data source and criteria

All PSDs from July 2005 to July 2020 were retrieved from the Australian Government web site dedicated to PBAC outcomes (18). The inclusion and exclusion of PSDs were based on the following three criteria: (i) Does the PSD report on a major submission? (We only included major submissions and not resubmissions or minor submissions to avoid duplication of clinical evidence). (ii) Does the submitted drug treat cancer? (Health technologies, such as cell therapies, devices, and gene therapies, were excluded). (iii) Does the PSD provide sufficient clinical evidence for extraction?

**Figure 1.**
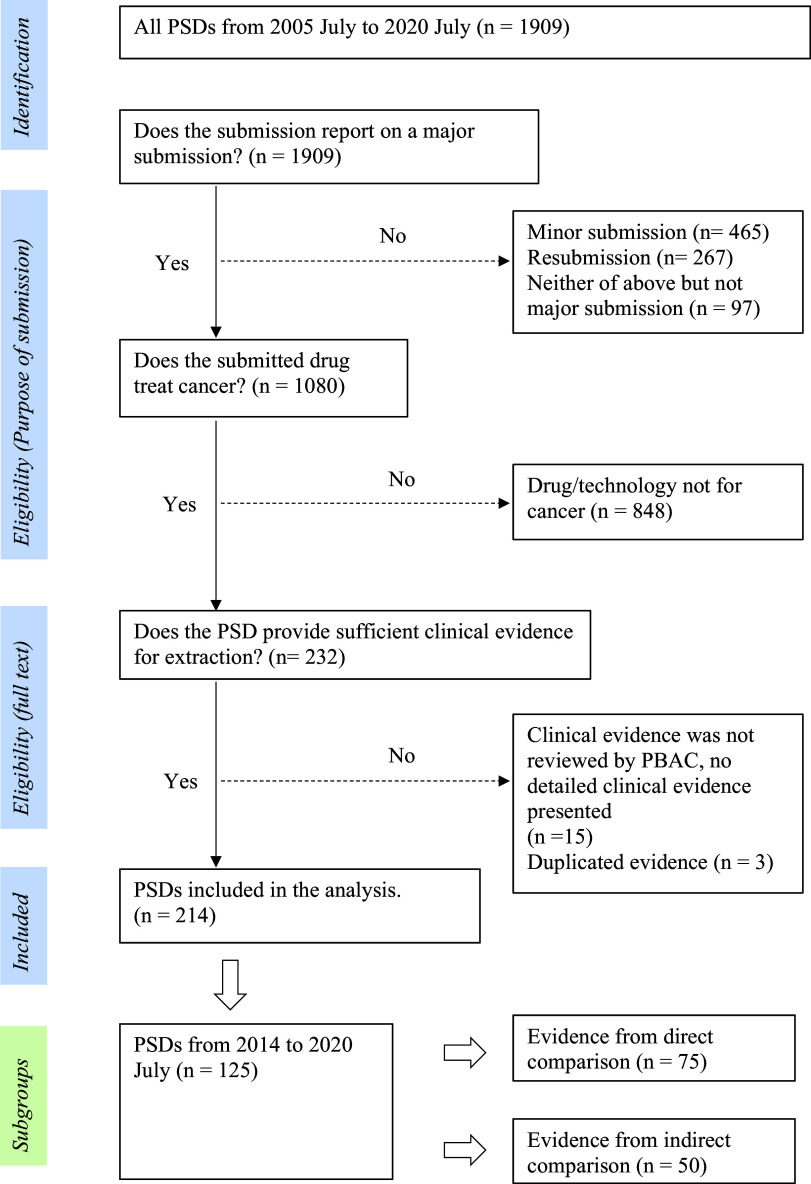
Identification and selection of evidence

Details of exclusions and inclusions are provided in Figure 1. Y.G. reviewed the PSDs against inclusion/exclusion eligibility and checked these with T.M. Y.G. extracted data from all included PSDs. Independent validation of the data extracted and the data extraction process was undertaken by MLK. This was undertaken through stratified random sampling of 20 percent of PSDs published each year. This approach was used as there may have been a variance in interpretation of PSD data due to changes in PBAC processes and guidelines over time (14). The sampled PSDs were extracted by M.L. Any disagreements were resolved through discussion by all three authors. Due to the complexity of clinical evidence presented in PSDs, we only reviewed the evidence considered pivotal in underpinning the clinical claims from the sponsor and the PBAC decision (details are in Supplementary Material S1). We extracted the key head-to-head or indirect evidence that was the basis of the clinical effectiveness claim. When there were multiple studies, we prioritized the highest level of evidence. For example, if both direct (e.g., RCTs) and indirect comparison evidence were presented, the direct evidence was extracted. If both RCT and single-arm studies were presented, the RCT was extracted as this would likely have the greatest influence on PBAC decision making and judgement of study quality.

### Variables

Due to several changes being made in the format of PSDs since they first became available in 2005, the selection of variables was dependent on the availability of data across the different time periods. The changes observed in PSDs from 2005 to 2020 are provided in Supplementary Material S2. We used data from 2014 to 2020 for the regression analysis because it was when all the variables in this study were reported on.

### Dependent variable and proxies for evidence quality

1) Risk of bias:

For direct evidence:

The instrument used to assess RoB for RCTs changed slightly over time (14). Before 2016, the assessment of RoB focused on three main components, (i) randomization, (ii) blinding, and (iii) follow-up. After 2016, the RoB assessments were based on the Cochrane RoB tool, which included domains such as random sequence generation, allocation concealment, blinding (participants and personnel, outcome assessment), incomplete outcome data, selective reporting, and other bias. Although these domains were explicitly addressed after 2016, prior to 2016, these domains were assessed but grouped simply under randomization, blinding, and follow-up.

We extracted RoB judgements made on the key head-to-head trials from the PSDs. In our study, RoB for direct comparisons in the PSDs was categorized as either “low” or “moderate/high/unclear.”

For indirect evidence:

The RoB assessment was based on whether the transitivity assumption was met or not. Earlier PSDs used the terms “comparability” or “exchangeability” instead of transitivity, although all referred to the same concept of similarity between the study arms being indirectly compared.

In our study, if there was any mention of violation of the transitivity assumption in the PSDs, or there was mention of an imbalance in patients’ baseline characteristics between study arms being compared, the transitivity assumption was considered to be not met.

2) Directness of the evidence: Evidence was categorized as “direct” or “indirect” in our study. An indirect comparison can compare the drug of interest with its comparator by way of a common reference arm or without a common reference arm.

3) Study design of the proposed cancer drugs: The study designs were categorized as “RCT” or “single arm or observational studies (SA/OB studies).” The inclusion of a comparator is critical for determining the incremental benefit of a new medicine and the findings from observational studies are likely to be affected by confounding.

4) Sample size: Smaller studies are likely to have an imbalance in prognostic factors or treatment effect modifiers between trial arms, potentially biasing the findings.

### Classification of cancer drugs

Drugs were classified by the first three digits of Anatomical Therapeutic Chemical code (ATC code) developed by World Health Organization (19).

### Independent variables

To evaluate the quality of evidence over the last 15 years, the year of PSD publication was extracted as a continuous variable. Other variables that we believed could independently affect the study quality or study quality over time were the maturity of overall survival (OS) data in a trial, disease rarity, whether the drug was first to market, participation in the TGA–PBAC parallel process and whether it was a codependent technology.

The maturity of OS data is used as an indicator of likely clinical benefit (20). The effectiveness of cancer medicines is generally bench-marked on the OS benefits that are obtained. OS can take some time to determine, and so trial results are sometimes presented that are immature, with insufficient patient follow-up. OS was considered of reasonable maturity when median OS was reached in the clinical trials supplied as key evidence.

The TGA regulatory definition of rare disease was utilized, which defines a disease as rare when it affects not more than 5 in 10,000 individuals (21). This is consistent with the definition used by the internationally recognized Orphanet database (22), so we used this database to classify all of the individual cancer indications.

Another potential determinant of the quality of evidence was the sequence of presentation to the market. “Me-too” drugs have a similar mechanism of action to established drugs with minor modifications (23). Nearly parallel development of new classes of chemical entities is common (23). Thus, there might be no comparator in the same class for both the first entry and the drug second to the market in their phase III clinical studies. This is challenging as, when the first entry is listed on the PBS, the drug second to the market must provide evidence against the first-in-class medicine to prove comparative effectiveness. Instead of head-to-head RCTs, which are expensive and can take several years to conduct, indirect comparisons between the drug second to the market and the first-in-class via a common comparator can be a more effortless and cheaper option (24). The drug mechanism of action was defined using the database of Kyoto Encyclopedia of Genes and Genomes (25). Identification of drugs second to the market was determined by chronological order (details are in Supplementary Material S3).

### Statistical analysis

Trial sample size and year of submission were continuous variables. The rest of the variables, including the remaining proxies for the quality of evidence and independent variables, were categorized as binary. Continuous variables were summarized by mean and standard deviations; categorical variables were summarized by frequency and percentage. Univariate logistic regression was adopted to screen independent variables that were associated with the quality of evidence or changes in evidence quality over time. The time trend of the change in the quality of evidence was determined by a multivariate logistic regression model, adjusted by variables showing associations in the univariate analysis. The time trend for sample size was determined by linear regression. Due to the availability of data, analyses were conducted within four subgroups by different time periods, so the time trend of quality of evidence was explored over four different time periods, 2005 to 2020, 2014 to 2020, 2014 to 2020 (only for direct comparisons), and 2014 to 2020 (only for indirect comparisons). The analysis of categorical variables was provided as an odds ratio (OR) for effect estimation with a 95 percent confidence interval (95% CI). For continuous variables, *R* ^2^, estimated regression coefficients (β), and 95% CI were given. *P* values were presented. Data extraction was performed in Microsoft Excel 2016 and data analyzed using R version 4.1.0.

## Results

**Figure 2.**
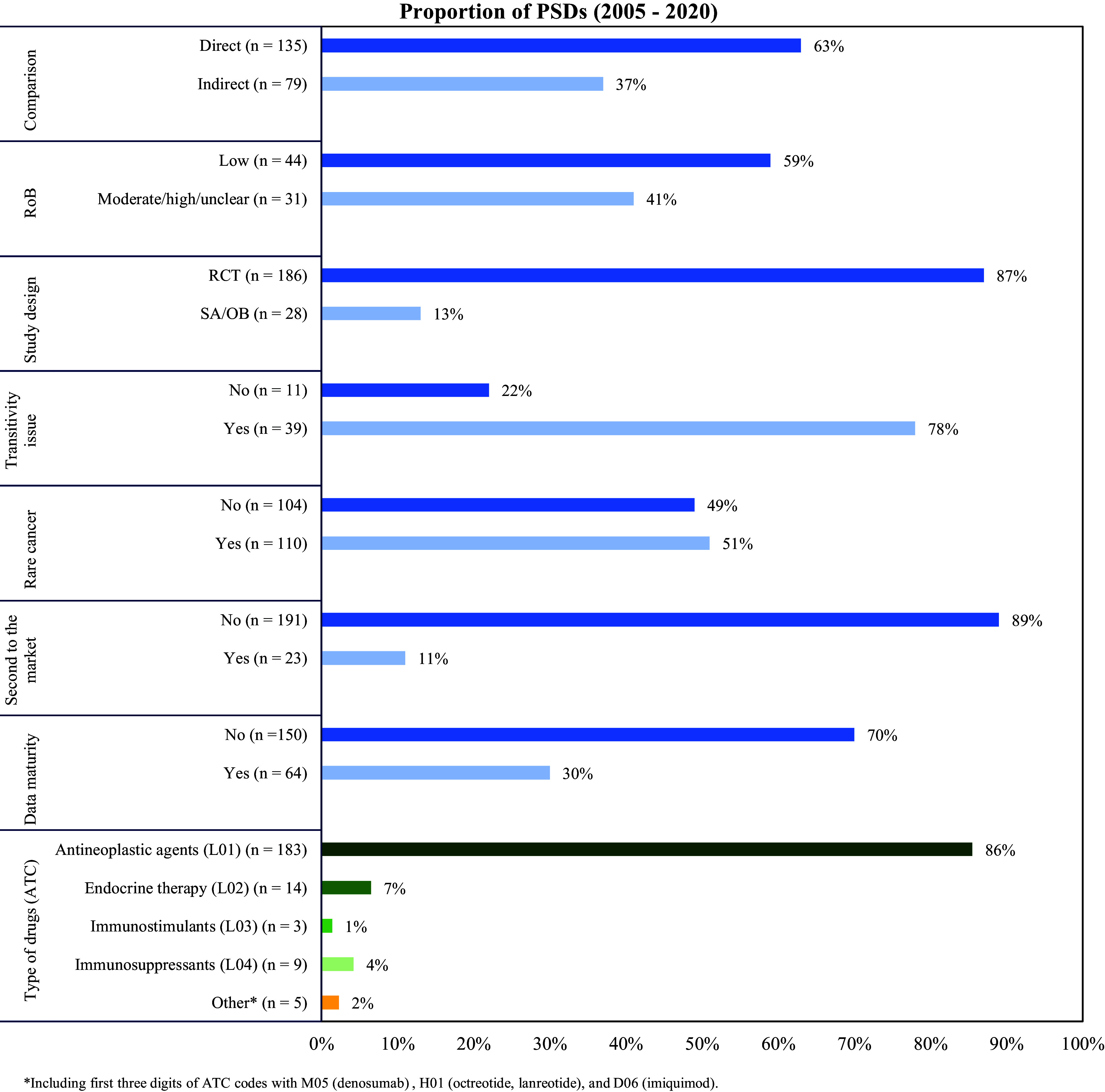
Reported characteristics of key evidence in Public Summary Documents concerning cancer medicines (2005-2020)

From July 2005 to July 2020 (Figure 1), a total of 214 PSDs were included in the analysis. Of these 214 PSDs, 63 percent presented clinical evidence containing a direct comparison and 37 percent (*n* = 79) discussed submissions that lacked direct comparative evidence (Figure 2). The majority of proposed cancer drugs were supported by RCTs (87 percent, *n* = 186), and 13 percent (*n* = 28) by SA/OB studies (Figure 2). Most of the proposed medicines were antineoplastic agents (86 percent, *n* = 184), followed by endocrine therapy (7 percent, *n* = 14), and immunosuppressants (4 percent, *n* = 9). In terms of certainty about the comparative clinical benefit, only 30 percent of PSDs (*n* = 64) reported on submissions that presented comparative OS data that was reasonably mature (Figure 2). Fifty-one percent of PSDs (*n* = 110) reported on submissions for medicines targeting rare cancers, and twenty-three PSDs (11 percent) reported outcomes for drugs second to the market (Figure 2).

Since 2014, information on the parallel process, codependent technology submissions, and sample size of clinical evidence was routinely reported. From March 2014 to July 2020, 125 PSDs were included in the analysis. Among these 125 PSDs, seventy-five reported pivotal evidence that involved a direct comparison, whereas fifty PSDs used indirect comparative evidence (Table 1). Forty-one percent (*n* = 31) of PSDs reported direct comparative evidence that had a moderate/high/unclear RoB and 78 percent (*n* = 39) of PSDs reported indirect comparisons that failed to meet the transitivity assumption (Table 1). The mean ( 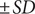 ) sample size for RCT pivotal evidence was 555 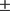 381 and for single-arm studies was 120 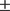 70. Sixty-one PSDs (49 percent) published between 2014 and 2020 discussed medicine submissions utilizing the TGA–PBAC parallel process, but the proportion of PSDs concerning codependent submissions remained low (*n* = 13, 10 percent) (Table 1).

**Table 1.** Changes in evidence base characteristics over time (2014-2020)

		2020		2019		2018		2017		2016		2015		2014		Total	
Year		*n*	%	*n*	%	*n*	%	*n*	%	*n*	%	*n*	%	*n*	%	*n*	%
Total		12	100	24	100	21	100	18	100	19	100	18	100	13	100	125	100
Direct comparison	Total	7	58	13	54	10	48	10	56	14	74	11	61	10	77	75	60
	RoB (moderate/high/unclear)	5	71	5	38	3	30	7	70	5	36	4	36	2	20	31	41
Indirect comparison	Total	5	42	11	46	11	52	8	44	5	26	7	39	3	23	50	40
	Transitivity concerns	2	40	7	64	11	100	7	88	4	80	6	86	2	67	39	78
Rare cancer	Total	5	42	11	46	13	62	9	50	11	58	12	67	6	46	67	54
	Direct	3	43	8	62	5	50	5	50	9	64	8	73	4	40	42	56
	Indirect	2	40	3	27	8	73	4	50	2	40	4	57	2	67	25	50
Second to the market	Total	3	25	4	17	4	19	2	11	1	5	1	6	0	0	15	12
	Direct	0	0	0	0	1	10	0	0	1	7	0	0	0	0	2	3
	Indirect	3	60	4	36	3	27	2	25	0	0	1	14	0	0	13	26
Data maturity	Total	1	8	10	42	8	38	3	17	11	58	2	11	2	15	37	30
	Direct	1	14	6	46	4	40	2	20	8	57	1	9	2	20	24	32
	Indirect	0	0	4	36	4	36	1	13	3	60	1	14	0	0	13	26
Parallel process	Total	4	33	10	42	12	57	10	56	7	37	11	61	7	54	61	49
	Direct	2	29	6	46	7	70	6	60	7	50	6	55	4	40	38	51
	Indirect	2	40	4	36	5	45	4	50	0	0	5	71	3	100	23	46
Codependent submission	Total	3	25	3	13	2	10	3	17	2	11	0	0	0	0	13	10
	Direct	2	29	2	15	0	0	1	10	2	14	0	0	0	0	7	9
	Indirect	1	20	1	9	2	18	2	25	0	0	0	0	0	0	6	12

In Table 2, univariate analyses were conducted to detect trends over time. From 2014 to 2020, there was a 51 percent and 47 percent increase in odds per year, respectively, of a submission being a drug that was second to the market (OR 1.51, 95% CI: 1.10, 2.21) or a codependent technology submission (OR 1.47, 95% CI: 1.05, 2.19). Variables that were associated with the quality of evidence included whether the drug was second to the market, treated a rare cancer, or had sufficient OS data maturity.

**Table 2.** Univariate analysis of relationship between evidence characteristics (independent variable subgroups) and markers of evidence quality, by different time periods and study type

	Years vs. independent variables				Independent variables vs. proxy of quality of evidence															
	Year				Indirect comparison				Study design to be SA/OB				RoB to be moderate/high/unclear				Transitivity issue			
Subgroups	*N*	%a	OR 95% CI	*p*	*N*	%b	OR 95% CI	*p*	*N*	%b	OR 95% CI	*p*	*N*	%b	OR 95% CI	*p*	*N*	%b	OR 95% CI	*p*
*2005–2020*																				
Total	214	.	.		79	.	.		28	.	.		.	.	.		.	.	.	
Second to the market	23	11	1.07 (0.97, 1.20)	0.20	17	22	5.90 (2.32, 17.01)	0.00	2	7	0.60 (0.09, 2.23)	0.51	.	.	.		.	.	.	
Rare cancer	110	52	1.01 (0.95, 1.08)	0.62	44	56	1.31 (0.75, 2.30)	0.34	21	75	3.27 (1.38, 8.64)	0.01	.	.	.		.	.	.	
Data maturity	64	30	1.00 (0.94, 1.07)	0.89	20	25	0.70 (0.357, 1.29)	0.26	4	14	0.35 (0.10, 0.96)	0.06	.	.	.		.	.	.	
																				
*2014*–*2020*																				
Total	125	.			50				20											
Second to the market	15	12	1.51 (1.10, 2.21)	0.02	13	26	12.82 (3.31, 84.81)	0.00	1	5	0.34 (0.02, 1.87)	0.31	.	.	.		.	.	.	
Rare cancer	67	54	0.92 (0.76, 1.11)	0.39	25	50	0.79 (0.38, 1.61)	0.51	13	65	1.75 (0.66, 5.00)	0.27	.	.	.		.	.	.	
Data maturity	37	30	1.08 (0.88, 1.33)	0.48	13	26	0.75 (0.33, 1.64)	0.47	3	15	0.37 (0.08, 1.19)	0.13	.	.	.		.	.	.	
Codependent	13	10	1.47 (1.05, 2.19)	0.04	6	12	1.32 (0.40, 4.24)	0.63	3	15	1.68 (0.35, 6.17)	0.47	.	.	.		.	.	.	
Parallel process	61	49	0.90 (0.74, 1.09)	0.28	23	46	0.83 (0.40, 1.70)	0.61	11	55	1.34 (0.51, 3.59)	0.54	.	.	.		.	.	.	
*2014*–*2020 direct comparison (ALL RCT)*																				
Total	75	.											31	.	.		.	.	.	
Second to the market	2	3	1.03 (0.46, 2.33)	0.93	.	.	.		.	.	.		0	0	.	.	.	.	.	
Rare cancer	42	56	0.97 (0.76, 1.24)	0.81	.	.	.		.	.	.		20	65	1.82 (0.72, 4.77)	0.21	.	.	.	
Data maturity	24	32	1.10 (0.86, 1.44)	0.45	.	.	.		.	.	.		6	19	0.35 (0.11, 0.98)	0.05	.	.	.	
Codependent	7	9	1.52 (0.98, 2.61)	0.08	.	.	.		.	.	.		4	13	2.02 (0.42, 10.96)	0.38	.	.	.	
Parallel process	38	51	0.98 (0.77, 1.24)	0.86	.	.	.		.	.	.		13	42	0.55 (0.21, 1.38)	0.21	.	.	.	
*2014–2020 indirect comparison (both RCT and SA/OB included)*																				
Total	50	.			.	.	.		.	.	.		.	.	.		39	.	.	.
Second to the market	13	26	1.71 (1.11, 2.92)	0.03	.	.	.		.	.	.		.	.	.		8	21	0.31 (0.07, 1.31)	0.11
Rare cancer	25	50	0.85 (0.61, 1.17)	0.33	.	.	.		.	.	.		.	.	.		22	56	3.45 (0.85, 17.62)	0.10
Data maturity	13	26	1.06 (0.74, 1.56)	0.74	.	.	.		.	.	.		.	.	.		11	28	1.77 (0.38, 12.82)	0.51
Codependent	6	12	1.39 (0.82, 2.66)	0.26	.	.	.		.	.	.		.	.	.		6	15	.	.
Parallel process	23	46	0.79 (0.55, 1.08)	0.14	.	.	.		.	.	.		.	.	.		20	51	2.81 (0.70, 14.30)	0.17

*Note:* %a, percentage of PSDs in individual subgroups in year range; %b, percentage of PSDs with independent variable amongst total PSDs. For example, there are 22 percent of PSDs concerning a drug second to the market among all indirect comparisons from 2005 to 2020.

Table 3 presents the multivariate analysis, which demonstrates the time trends in the quality of evidence. In the unadjusted model, there was a 22 percent increase in odds per year of having moderate/high/unclear RoB in clinical evidence with a direct comparison (OR 1.22, 95% CI: 0.95, 1.57). After adjusting for data maturity and rare indications which were independently associated with RoB, the likelihood of direct clinical evidence having a moderate/high/unclear RoB firmed to a 30 percent increase in odds per year since 2014 (AOR 1.30, 95% CI: 0.99, 1.70). There was no change in the quality of evidence over time when quality was represented independently in terms of the directness of evidence, study design, transitivity assumption being met, and sample size (Table 3).

**Table 3.** Predictors of evidence quality over time (multivariate analysis)

	Independent variables (*Y*)			
Range of model	Proxy of quality of evidence	Years(*X*) + covariates	OR/AOR^a^/β^b^ 95% CI	*p* and *R* ^2^^b^
Model 1 (2005–2020)	Indirect comparison	Year	1.05 (0.98, 1.12)	0.12
		Year + second to the market	1.04 (0.97, 1.11)	0.24
Model 1′ (2014–2020)	Indirect comparison	Year	1.17 (0.96, 1.42)	0.13
		Year + second to the market + codependent technology	1.07 (0.86, 1.32)	0.56
Model 2 (2005–2020)	SA/OB studies	Year	1.04 (0.95, 1.15)	0.35
		Year + rare cancer + data maturity	1.05 (0.95, 1.16)	0.35
Model 2′ (2014–2020)	SA/OB studies	Year	0.89 (0.68, 1.15)	0.37
		Year + rare cancer + data maturity	0.91 (0.70, 1.19)	0.50
Model 3 (2014–2020) (direct)	RoB (moderate/high/unclear)	Year	1.22 (0.95, 1.57)	0.12
		Year + data maturity + rare cancer	1.30 (0.99, 1.70)	0.07
Model 4 (2014–2020) (indirect)	Transitivity issue	Year	0.76 (0.48, 1.13)	0.21
		Year + second to the market + rare cancer	0.88 (0.53, 1.40)	0.6
Model 5 (2014–2020)	Sample size of RCT	Year	−7.29 (−45.12, 33.53)	0.72, 0.00
	Sample size SA/OB	Year	−3.12 (−21.73, 15.49)	0.68, 0.01

a Adjusted OR by variables listed on the left.

b Only for sample size.

## Discussion

Our main finding was that the reporting of RoB as moderate/high/unclear in head-to-head trials increased by 30 percent per year, after adjusting for data maturity and rare indications. Although these results are statistically insignificant, which might be due to the smaller number of PSDs in the later time period (2014–2020), the effect estimate, and confidence interval suggests an important decline in the certainty of the evidence over time. As the other variables that we investigated that were associated with quality of evidence did not deteriorate over time, it is unclear what specific factors are driving this decline. Many factors could have contributed to this decline in quality, such as increases in cross-over/treatment switching between trial arms, the use of open-label trials in conjunction with subjective outcomes, composite primary outcomes, and incomplete outcomes due to censoring or loss of follow-up. Unfortunately, data on these factors were not routinely reported in the PSDs over the past 15 years. Future follow-up studies are needed to discover the factors contributing to this trend.

The high and increasing proportion of clinical evidence with poor internal validity is a concern. We found transitivity issues were mentioned in 78 percent of PSDs where indirect comparison evidence supported the clinical claim, and a concerning level of RoB mentioned in 41 percent of PSDs where head-to-head data supported a clinical claim (Figure 2). These findings are consistent with previous studies conducted in Australia, Europe, and Canada. For instance, Naci and colleagues found that RCTs supported most cancer drugs approved by European Medicines Agency (EMA), but almost half of these submissions provided low certainty of clinical outcomes due to a high RoB (6). Jenei et al. (26) reviewed three recent years’ oncology drug submissions to the pan-Canadian Oncology Drug Review and found that submissions had questionable clinical trial validity, which resulted from the selection bias, reporting bias, performance bias, and attrition bias in the clinical evidence. In Australia, Wonder and colleagues found that the most significant issue in submissions to PBAC from 2005 to 2012 was the determination of a medicine’s comparative performance in the proposed population (13), which was determined by a wide range of factors such as poor-quality evidence, use of surrogate outcomes, or availability of RCT evidence (13). The high RoB and lower quality of evidence contribute to uncertainty in the clinical benefit (10). Despite this international concern about the quality of drug submissions this evidence continues to be submitted and, from our research findings, appears to be deteriorating over time. Which begs the question, is this increase in poor quality evidence affecting the rate of funding rejections over time? And, if not, does this mean that the quality control mechanisms put in place to protect the health of the public are eroding?

Our study found that only a small proportion of cancer drug submissions had OS data that had reached maturity (30 percent). Data immaturity in oncology trials has become common, and this poses a challenge for decision making for both market authorization (7;10) and reimbursement (11;20). In addition, to achieve an early submission to regulatory and HTA bodies, pharmaceutical companies may pursue clinical endpoints that take less time to follow up, such as progression-free survival. However, evidence has shown that such surrogate outcomes frequently fail to translate into real clinical benefit (8;9). In the UK, between 2010 and 2016, the Cancer Drug Fund (CDF) reimbursed high-cost cancer drugs that were appraised but not approved by National Institute for Health and Care Excellence (11). Subsequently, most of the drugs funded by the CDF were unable to demonstrate meaningful clinical improvement in survival, quality of life, or decreases in toxicity. As a result, they were eventually delisted from the CDF after a short period of time (11).

We found that the odds of personalized medicines (codependent technologies) being submitted to PBAC increased by almost 50 percent from 2014 to 2020, indicating the impact of pharmacogenetics in oncology over the last decade. We also observed an increase in submissions of drugs that are second to the market (OR 1.47 95% CI: 1.05, 2.19), which is consistent with the findings of Tito and colleagues who demonstrated that more pharmaceutical companies have pursued cancer drugs with marginal incremental benefit over existing treatments in recent decades (27). Unsurprisingly, in our univariate analysis, we observed a significant correlation in the PSDs between drugs that are second to the market and an evidence base consisting of indirect comparison. The association confirms that the estimated magnitude of comparative clinical benefit is more likely to be uncertain for the drug second to the market because the pivotal evidence lacks randomization. PBAC allows a second-to-market drug to be reimbursed with equivalent cost against the first entry if non-inferior efficacy is demonstrated (14).

Our study included PSDs published in the past 15 years. This was the first study to evaluate the quality of clinical evidence for oncology medicines, as determined by the PBAC, when considering submissions for reimbursement in Australia. In addition, this study addressed the impact of a range of factors over time on this quality of evidence, including the maturity of clinical data, rare indications, and drugs second to the market, utilization of the TGA–PBAC parallel process, and the codependent technology pathway. The study had several limitations: (i) We could not report all analyses over the full 15 years’ time period due to the lack of available data. This might have underpowered some of our analyses. (ii) There might be a small amount of information loss concerning data maturity because median OS was sometimes redacted in the PSDs. It is also acknowledged that updated OS data were often provided by sponsors in subsequent submissions to the PBAC. Therefore, the proportion of resubmissions providing mature median OS data would be higher than in our study. (iii) Although 20 percent of PSDs in each year were validated independently by a second researcher (M.L.), and checked for discrepancies by a third researcher (T.M.), much of the data were extracted by a single person (Y.G.), which can increase the risk of random error.

This paper highlights our concerns regarding the quality of evidence for cancer drugs proposed for funding in Australia and points to future research that might be able to address this: (i) Our study found that the RoB in clinical evidence in oncology submissions was a major concern of the PBAC in nearly half of the submissions received. The early engagement of HTA agencies during the drug development process, especially providing scientific advice regarding choice of comparator, study design, and endpoints or outcomes, might help manufacturers to conduct rigorous and adequate clinical trials that meet the requirements of both regulators and HTA appraisal committees such as PBAC. Further research is needed to detect the drivers of this judgement in order to assist manufacturers in the design of oncology trials and compilation of evidence that is fit-for-purpose for funding decision making. (ii) There was a high proportion of PSDs that reported a lack of mature data in the evidence base. Post-launch studies are urgently needed to monitor the survival benefits associated with newly listed cancer drugs, along with a re-assessment of the value for money of these drugs – a lifecycle HTA approach. (iii) Our findings show that the transitivity assumptions of indirectly compared studies are frequently not met and that these indirect comparisons predominantly concern “me-too” later to the market drugs. This suggests there is uncertainty whether these later-to-the-market drugs are in fact non-inferior. Research is needed to monitor the performance of medicines publicly funded on the basis of indirect comparisons, to confirm or challenge these findings when later replicated by head-to-head studies.

## Conclusion

We found that funding decisions for cancer drugs in Australia are made in the context of significant uncertainty as the quality of clinical evidence provided is frequently poor and has deteriorated over the last decade. As the evidence dossiers submitted in Australia are reflective of dossiers submitted to other HTA bodies internationally, this poor-quality evidence contributes to a high degree of uncertainty in decision making, with opportunity costs to society and potentially consequent risks to patients.

## Supplementary material

For supplementary material accompanying this paper visit https://doi.org/10.1017/S0266462323000259. click here to view supplementary material
