# Supplementary material for: Is the quality of evidence in health technology assessment deteriorating over time? A case study on cancer drugs in Australia
Source: Int J Technol Assess Health Care. 2023 May 18;39(1):e28. doi: 10.1017/S0266462323000259 (PMC11574543; doi:10.1017/S0266462323000259)

Supplementary Files

Is The Quality Of Evidence in HTA Deteriorating Over Time? A Case Study on Cancer Drugs In Australia

Authors: Yuan Gao, MPH, Mah Laka, PhD, Tracy Merlin, PhD

Affiliation: Adelaide Health Technology Assessment, School of Public Health, University of Adelaide

Contact information for the corresponding Author:
Professor Tracy Merlin, PhD,
Head, School of Public Health;
Director, Adelaide Health Technology Assessment
School of Public Health,
University of Adelaide

Mail Drop DX650545
Adelaide, SA 5000
Australia
tracy.merlin@adelaide.edu.au
Phone: (61) 8 8313 3575

S 1 Decision rules for information extraction of cancer drug Public Summary Documents (PSDs)

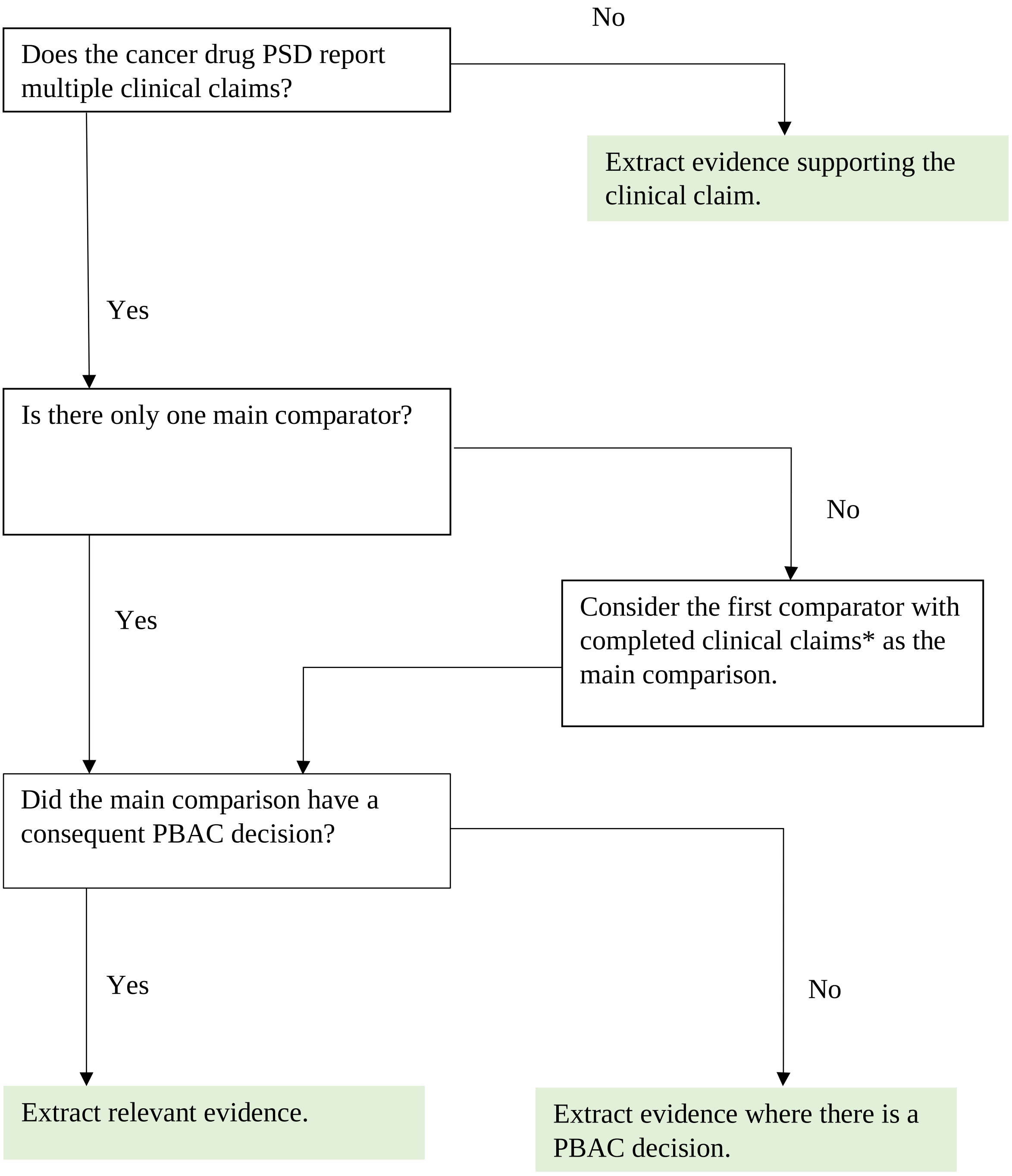

*Completed clinical claims means sponsors make clinical claims of comparative clinical effectiveness, safety, and cost-effectiveness between proposed medicine and comparator/s.

S 2 Changes in PSDs detected by our study

	2005 - 2013	2014 - 2020
Overall differences		
PSD number*	Not applicable	Applicable
Clarification of submission type (major, minor, resubmission, etc.) *	Not always clearly stated	Clearly stated in the purpose of submission
Co-dependent submission*	Not applicable	Applicable
Engagement of PBAC-TGA parallel process*	Not applicable	Applicable
Judgements of RoB for each trial from PBAC	Not available	Available
The sample size for each trial	Not available	Available
Judgements of maturity of data from PBAC*	Not available	Available
Clinical claims in terms of comparative clinical effectiveness and safety from sponsor*	Have missing information in clinical claims in comparative safety profile.	Available
Judgements toward clinical claims from PBAC	Have missing information in PBAC judgements, and the judgements were briefer than PSDs after 2014.	Detailed and available for all
Availability of trials' survival data*	Some of them were not available or redacted.	Most of them were available.
Only for evidence with indirect comparison		
Judgements toward adjustment methods of comparison from PBAC*	Not available	Available
Mentioned "naive comparison" if there is no common comparator*	Not available	Available
Judgements toward transitivity assumptions from PBAC*	Not available	Available

* Not strictly using 2014 as a cut-off; the range of cut-off lies between 2012 - 2014

S 3 Process of identification of drug second to market

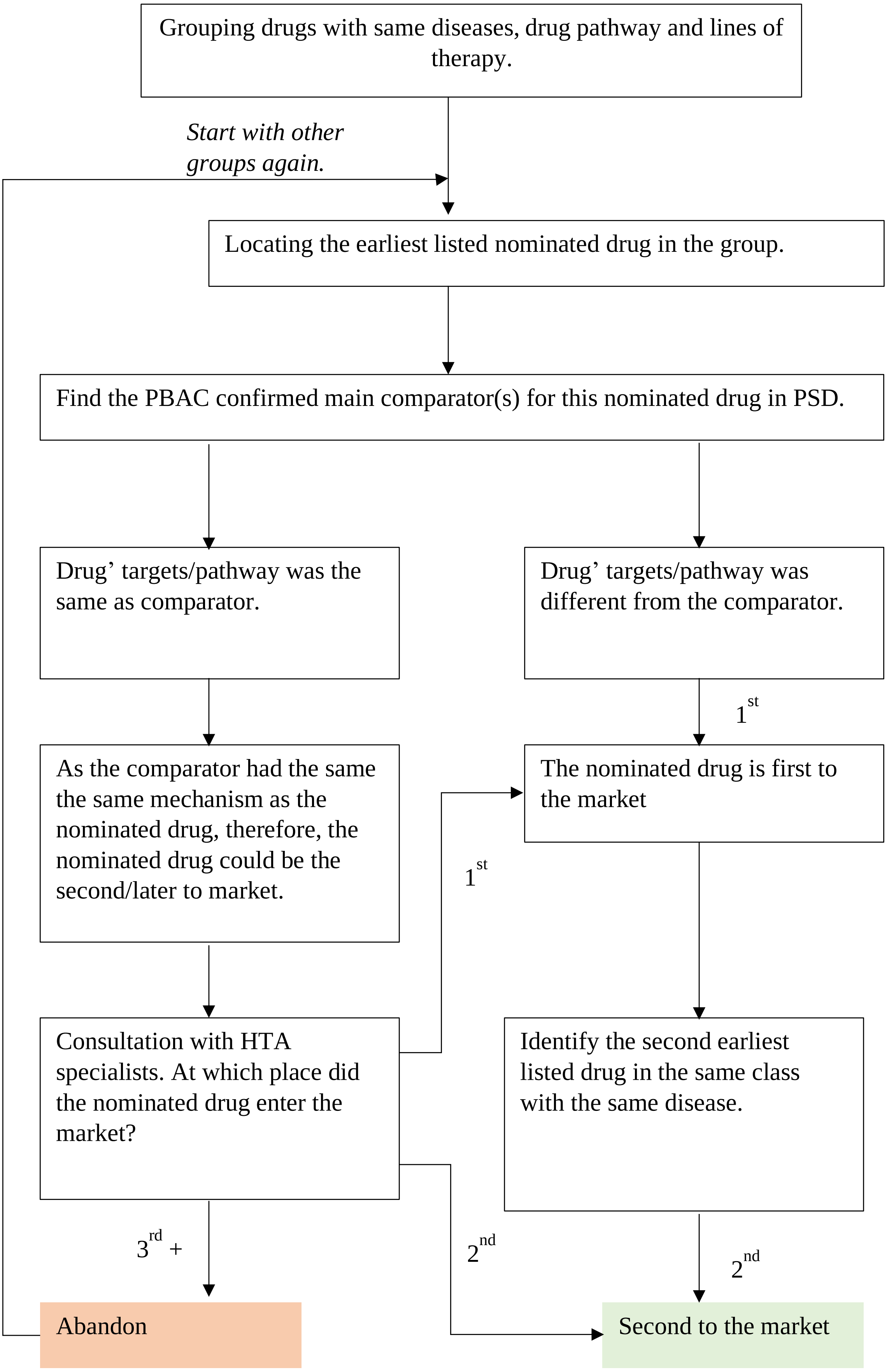

Supplement: Supplementary file 1 [file S0266462323000259sup001.pdf]
